# Endocardial role in arrhythmias induced by acute ventricular stretch and the involvement of Purkinje fibres, in isolated rat hearts

**DOI:** 10.1016/j.crphys.2023.100098

**Published:** 2023-02-01

**Authors:** Miriam Hurley, Sarbjot Kaur, Richard Walton, Amelia Power, Michel Haïssaguerre, Olivier Bernus, Marie-Louise Ward, Ed White

**Affiliations:** a School of Biomedical Sciences, University of Leeds, Leeds, UK; b Department of Physiology, University of Auckland, Auckland, New Zealand; c Université Bordeaux, INSERM Centre de recherche Cardio-Thoracique de Bordeaux, Pessac, Bordeaux, France; d IHU Liryc, Electrophysiology and Heart Modeling Institute, Fondation, Bordeaux Université, Pessac, Bordeaux, France; e Bordeaux University Hospital (CHU), Electrophysiology and Ablation Unit, Pessac, France

**Keywords:** Ventricular arrhythmias, Stretch, Endocardium, Purkinje fibres, TRPM4

## Abstract

Purkinje fibres (PFs) play an important role in some ventricular arrhythmias and acute ventricular stretch can evoke mechanically-induced arrhythmias. We tested whether PFs and specifically TRPM4 channels, play a role in these mechanically-induced arrhythmias. Pseudo-ECGs and left ventricular (LV) activation, measured by optical mapping, were recorded in isolated, Langendorff-perfused, rat hearts. The LV endocardial surface was irrigated with experimental agents, via an indwelling catheter. The number and period of ectopic activations was measured during LV lumen inflation via an indwelling fluid-filled balloon (100 μL added over 2 s, maintained for 38 s). Mechanically-induced arrhythmias occurred during balloon inflation: they were multifocal, maximal in the first 5 s and ceased within 20 s. Optical mapping revealed activation patterns indicating PF-mediated and ectopic focal sources. Irrigation of the LV lumen with Lugol solution (IK/I_2_) for 10s reduced ectopics by 93% (n = 16, P < 0.001); with ablation of endocardial PFs confirmed by histology. Five min irrigation of the LV lumen with 50 μM 9-Phenanthrol, a blocker of TRPM4 channels, reduced ectopics by 39% (n = 15, P < 0.01). Immunohistochemistry confirmed that TRPM4 was more abundant in PFs than myocardium. Our results show that the endocardial surface plays an important role in these mechanically-induced ectopic activations. Ectopic activation patterns indicate a participation of PFs in these arrhythmias, with a potential involvement of TRPM4 channels, shown by the reduction of arrhythmias by 9-Phenanthrol.

## Highlights

• The endocardial region plays an important role in acute stretch-induced arrhythmias/ectopics.
• The Purkinje fibre network is involved in acute stretch-induced ectopics.
• In rat, TRPM4 abundance is greater in Purkinje fibres than myocardium and a TRPM4 blocker attenuated stretch-induced ectopics.

## 1 Introduction

The electromechanical coupling of the heart is important in both normal and pathological function. The inter-dependence of mechanical and electrical activity is demonstrated by excitation-contraction coupling (Bers, 2002) and mechano-electric feedback/coupling (Orini et al., 2017; Quinn and Kohl, 2021). A manifestation of these interactions are mechanically-induced arrhythmias which occur in several clinical settings such as atrial fibrillation, *commotio cordis*, hypertrophy and heart failure e.g. (Quinn and Kohl, 2021; Kohl et al., 2011; Yamazaki et al., 2012).

A common experimental technique used to investigate responses to mechanical stimulation is acute cavity dilation/stretch in isolated hearts, using inflation of an indwelling fluid-filled balloon e.g. (Franz et al., 1992; Bell et al., 2011). Inflation of the balloon stretches the myocardium, increasing contractility via the Frank-Starling mechanism (Shiels and White, 2008). However, acute distension can also provoke mechanically-induced arrhythmias with the timing, duration and amplitude of stretch modulating the electrical response see (White, 2005). Arrhythmia presentation can vary from single ectopics coupled to single mechanical stimuli (Franz et al., 1992) to more complex profiles (Dick and Lab, 1998; Bode et al., 2001). The nature of the mechanical stimuli is difficult to characterise precisely, in addition to increased axial strain it may also include compression and shear. We use ‘mechanical stimulation’ and ‘stretch’ interchangeably to represent these collectively.

In isolated rabbit hearts (Quinn et al., 2017), showed that localised mechanical stimulation of the epicardial surface triggered arrhythmias that arose from the site of mechanical stimulation. If localised or regional stimulation is a common feature of mechanically-induced arrhythmias, then stimulation of the ventricular endocardial surface, by the inflation of an indwelling balloon, should be an important factor in the resultant arrhythmias. The initial aim of our study was to test this possibility.

At the ventricular endocardial surface are Purkinje fibres (PFs), the endothelium and the underlying endocardial myocardium. Each tissue has been reported to be sensitive to mechanical stimulation (Brutsaert et al., 1996; Kaufmann and Theophile, 1967; Kohl et al., 2011). Pertinently, the response of endothelial tissue to mechanical stimulation is paracrine in nature and slower than the electrical response associated with arrhythmias (Brutsaert et al., 1996). We used distinct structural and electrophysiological characteristics of PFs and the endocardial myocardium (Boyden et al., 2016; Haissaguerre et al., 2016) to investigate their relative contributions. Because of the thin structure and superficial endocardial location of surface PFs, brief exposure to Lugol solution (KI/I_2_) is a well-established technique for preferential chemical ablation of PFs (Damiano et al., 1986; Dosdall et al., 2008; Myles et al., 2012). An electrophysiological difference between PFs and ventricular myocardium is the greater abundance and influence of TRPM4 channels upon the electrical activity of PFs (Guinamard et al., 2015; Hof et al., 2016). TRPM4 channels are non-specific cation channels activated by Ca^2+^, 9-Phenanthrol (9-Phen) is a blocker of these channels (Guinamard et al., 2015). A further distinction is the broader and faster spread of electrical activation, in response to endocardial PF stimulation compared with endocardial myocardial stimulation (Martinez et al., 2018).

Although previous studies have shown isolated PFs to be mechanically sensitive (Kaufmann and Theophile, 1967; Dominguez and Fozzard, 1979), there has been relatively little study of PFs within the context of mechanically-activated arrhythmias. PFs lining the endocardial surface of the ventricles are stretched by chamber dilation (Canale et al., 1983). Thus, the mechanical activation of PFs is a plausible component of mechanically-induced arrhythmias (Ferrier, 1976). PFs are implicated in the induction and maintenance of multiple types of arrhythmias (Haissaguerre et al., 2016; Haïssaguerre et al., 2002) and PF ablation can be an effective treatment (Samo Ayou et al., 2014). Despite the importance of PFs to arrhythmias, mechanisms of action are not fully understood (Haissaguerre et al., 2016). Therefore, the investigation of the role PFs play in mechanically-induced arrhythmias is timely.

## 2 Materials and methods

### 2.1 Mechanically-induced arrhythmias

All animal studies conformed to the Guide for the Care and Use of Laboratory Animals. Male Wistar rats (N = 34, 250–310 g) were killed in accordance with local and national ethical regulations and approvals, University of Auckland AEC 001232; UK Home Office PPL No 70/8399. Hearts were rapidly removed and Langendorff perfused with a HEPES buffered physiological saline at 38 °C that contained (in mM): NaCl 142; KCl 6; MgSO_4_ 1.2; Na_2_HPO_4_ 1.2; HEPES 10, Glucose 10; CaCl_2_ 1.8 (pH 7.4). All chemicals were purchased from Sigma.

A fluid filled system, comprising a cellophane balloon connected via a thin polythene tube (outer diameter 0.9 mm) to a pressure transducer and syringe, was used to stretch the left ventricle (LV). The deflated balloon and a second tube (outer diameter, 0.9 mm) were placed in the LV lumen via the mitral valve and an opening created by the removal of part of the left atria. These tubes were secured in place by tying to the aortic cannula. The opening of the mitral valve and removal of left atrial tissue provided a route for LV drainage. Thus, the myocardium was Langendorff perfused with Tyrode's via the aorta and coronary circulation, while the tube in the LV lumen was used to irrigate the LV endocardial surface with experimental solutions. Needle electrodes were used to record pseudo-ECGs (negative electrode in the right atria, positive electrode in the ventricular apex, avoiding both ventricular lumens).

The deflated balloon was inflated until a stable pressure pulse of 15–20 mmHg was detected, at approximately 50 μL volume. This indicated the balloon was in contact with the LV endocardial surface and at the lower end of the Frank-Starling relationship. The ascending limb of the relationship was therefore available to accommodate stretching. Diastolic pressure was 15.4 ± 1.7 mm. The LV was stretched by inflation of the balloon with an additional 100 μL over 2 s. Balloon inflation was maintained for 38 s. The balloon was then deflated by withdrawing 100 μL. Thus, for each heart the LV was stretched to and from a constant volume. The inflation rate (50 μL/s) and final inflated volume of the balloon (150 μL) was consistent with those we have previously used to trigger mechanically-induced arrhythmias in hearts from similar sized animals (Benoist et al., 2014). These parameters were deemed appropriate for this study as they consistently provoked mechanically-induced arrhythmias, allowing us to investigate the phenomenon. The ability to repeat stretches in each heart indicated the tissue was not significantly damaged by stretching.

The endocardial surface was irrigated with Tyrode's solution or (i) 0.1 mL Lugol solution (120 mM KI, 38.6 mM I_2_) for 10 s followed by Tyrode's wash (ii) 3 mL of 50 μM 9-Phen for 5 min or (iii) Tyrode's. In each heart 2 stretches, at least 60 s apart (Dick and Lab, 1998) were performed following initial Tyrode's irrigation and 2 stretches following the intervention. The concentration of agents was based on previously reported effects.

Pseudo-ECG records were used to identify sinus and ectopic (non-sinus) activations. The number of ectopic activations caused by stretch was measured. The period of ectopic episodes was measured as the time from the first ectopic activation to the time of the first sinus beat, following the last ectopic. Ectopic activations were identified by their timing and waveform, relative to sinus rhythm activations. Data was analysed from 20 s of continuous records immediately before stretch and 20 s from the beginning of stretch. This 20 s period captured all ectopic activity.

Changes in pressure were used to indicate stretch, in the form of increased LV developed pressure (LVDP, an index of contractility). LVDP was the difference between diastolic and systolic pressures and was measured during steady state contractions at both 50 μL (17.1 ± 1.2 mmHg) and 150 μL (50.3 ± 3.7 mmHg) balloon volumes. Heart rate was calculated from R-R intervals of the pseudo-ECG (198 ± 7.4 bpm) mean ± SEM from 24 hearts. Heart rate, and LVDP, were measured to assess any changes in basal electrical and mechanical activity caused by pharmacological intervention. Data were acquired at 1 kHz and analysed with LabChart (ADInstruments, NZ). All data were collected in sinus rhythm, to preserve the physiological PF to myocardium activation sequence, at 38 °C.

Data were analysed with SPSS. Effects of interventions were tested by paired Student's t-test with the first and second stretches in Tyrode's paired with the equivalent stretch following an intervention. The term ‘significantly’ in the text refers to a statistically significant effect (P < 0.05).

### 2.2 Optical mapping of epicardial activation

The LV epicardial surface of Langendorff perfused hearts was optically mapped using RH237 (ThermoFisher). Motion artefacts were suppressed by perfusion of Blebbistatin (BioServ), dissolved in DMSO, given as a single 10 mL, 85 μM bolus in Tyrode's. RH237 was then given as an 8 mL, 75 μM bolus in Tyrode's. RH237 effluent was collected and re-introduced once. The Langendorff perfusion pump was stopped while bolus injections were given to prevent over perfusion of the heart. RH237 was excited by 530 nm light from 4 LEDs, emission light at 685 nm was collected by a Charge Coupled Device camera (Scimeasure) and appropriate filters (Cairn Research, UK) capturing at 1 kHz with an 80*80 pixel, 17.5 mm square field of view. Data were acquired with Turbo software (Scimeasure). Epicardial activation maps were created with BV analysis (Brainvision, Japan) using 3*3 spatial and cubic filters, maps were colour-coded with 3 ms contours. Epicardial activation was recorded in response to sinus activation, which involves conduction through the PF network; in response to focal endocardial myocardium stimulation by external Pt electrodes placed on the base of the LV; and in response to balloon inflation as previously described. Pseudo-ECGs were recorded by non-contact electrodes in the re-circulating, heated experimental chamber, into which the heart was immersed for imaging.

### 2.3 Histology, identification of necrotic tissue following Lugol treatment

Triphenyltetrazolium Chloride (TTC; Merck, T8877) was used to identify the presence of necrotic tissue after the perfusion of Lugol solution e.g. (Myles et al., 2012). A stock solution (29.87 mM TTC, 135.15 mM Trizma-HCl, 69.34 mM Trizma base) was warmed to 37 °C prior to use. After the LV lumen was irrigated with 0.1 mL Lugol solution over 10 s followed by 0.9 mL Tyrode's, the heart was dissected to reveal the endocardial surface and transmural cut edges of the left and right ventricle. The heart was immersed in the TTC stock solution for 40 min followed by fixation in formalin solution for 60 min both at 20–22 °C. Images were acquired using a Sony cyber-shot digital camera, with healthy muscle exhibiting a dark red appearance contrasting with the white appearance of necrotic tissue.

### 2.4 Histology, immunofluorescent labelling of connexin 40

High levels of connexin 40 (Cx40), relative to the myocardium, were used as an indicator for PFs (Atkinson et al., 2011, 2013). Upon completion of functional experiments, whole hearts were fixed in 1% paraformaldehyde (PFA) for 60 min at 4 °C, washed with phosphate buffered saline (PBS, containing in mM KH_2_PO_4_ 1.06; NaCl 155; Na_2_HPO_4_–7H_2_0 2.97) for a minimum of 60 min and exposed to a rising 10–30% sucrose gradient. Tissue was snap frozen in liquid nitrogen, with the use of isopentane. Tissue sections, 10 μm in width, were affixed to 0.05% poly-L-lysine coated #1.5H coverslips and stored at −20 °C until use.

At 20–22 °C, tissue sections were hydrated with PBS prior to the application of Image-iT Signal Enhancer (Life Technologies) for 60 min as a blocking buffer. A primary antibody was diluted in incubation solution (w/v or v/v; 0.05% NaN_3_, 2% bovine serum albumin, 2% normal goat serum and 0.05% Triton X-100) and incubated overnight at 4 °C. Dilution was dependent upon the required antibody working concentration to label Cx40 (1:200; Invitrogen, 36–4900). Afterwards, tissue was washed 3 times in PBS for 20 min each, before the application of a secondary antibody for 2 h at 20–22 °C, which was diluted in incubation solution (Alexa 488; 1:200; Invitrogen, A11008). Tissue was washed 3 times in PBS for 20 min each. To stain the nuclei, 1 μg/mL DAPI (Sigma-Aldrich, D9542) was applied for 5 min and tissue washed a final 3 times in PBS for 5 min each prior to imaging.

An Evos Cell Imaging System, using the DAPI (excitation: 357/44 nm; emission: 447/60 nm) and GFP (excitation: 470/22 nm; emission: 510/42 nm) filter system, was utilised. Using ImageJ (Fiji), a 50-pixel sliding paraboloid was applied to each acquisition channel to subtract background fluorescence. The gray value of Cx40 label was used as a measure of signal intensity, relative to pixel value across the complete dataset and presented as mean ± SEM. Statistical analysis of data was performed in SPSS, with different regions of the tissue section compared using one-way ANOVA.

### 2.5 Histology, immunofluorescent labelling of TRPM4

Following removal, hearts were immediately immersed in ice-cold 20 mM BDM with 10 mM glucose in Krebs-Henseleit buffer. PFs were isolated with a small piece of LV myocardial wall and fixed with 2% PFA for an hour at 4 °C.

Samples were incubated for 60 min with iT FX signal enhancer (Life Technologies) and washed twice for 5 min each with PBS. Tissue was incubated overnight at 4 °C with primary antibody rabbit anti-TRPM4 (1:100; Invitrogen, PA5-77324) in PBS with 1% BSA, 0.01% sodium azide. The following day, tissue was given 3 × 5 min washes each with PBS prior to the addition of secondary antibody (Alexa 594; 1:200, Thermofisher Scientific). Tissue was incubated for 2 h at room temperature with secondary antibody and followed by 3, 5 min washes with PBS before mounting with Prolong Gold (Thermofisher Scientific) on a glass slide. Two-dimensional images were acquired using an FV1000 Olympus confocal microscope. The integrated density and mean gray value of TRPM4 label was measured from myocardium and PFs (n = 3 hearts) using Image J-(Fiji), and presented as the mean integrated density ± SEM. Statistical analysis of data was performed in GraphPad Prism 7.03, and differences were compared using paired t-tests.

## 3 Results

### 3.1 Acute stretch-induced ectopics and their attenuation by Lugol

**Fig. 1:**
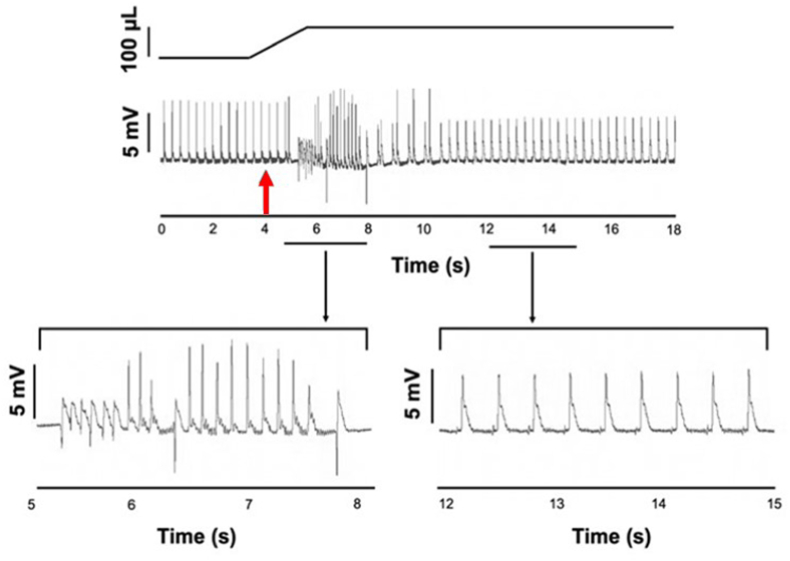
Acute left ventricular stretch induces ectopics. Upper trace, schematic change in balloon volume, and change in pseudo-ECG in a Langendorff perfused, isolated rat heart in sinus rhythm. The red arrow, at approximately 4 s, indicates the onset of LV stretch by injection of 100 μL into a balloon placed in the LV lumen. Mechanically induced ectopics began during the increase in luminal volume, with a spontaneous return to sinus rhythm at approximately 11 s. Ectopic activations were identified by their timing and waveform compared to sinus configurations. Lower trace, expanded time scale of the pseudo-ECG trace highlighting a period of ectopic activations (between 5 and 8 s) and during the return to sinus rhythm (between 12 and 15 s). Ectopic activations had varied waveforms including negative and positive going initial vectors while regular P-R waveforms are visible in the sinus trace. . (For interpretation of the references to colour in this figure legend, the reader is referred to the Web version of this article.)

In isolated, Langendorff perfused rat hearts, LV stretch by balloon inflation generated ectopic activations that caused a disruption of the rhythmic pattern of the pseudo-ECG (Fig. 1). Ectopic excitations manifested several morphologies with both + ve and –ve initial vectors. Fig. 1 demonstrates single ectopics, brief tachycardia and doublets. Episodes of ectopic activity began as balloon volume was increased and were most frequent in the ensuing 5s. Sinus rhythm spontaneously returned in all preparations within 20s of the start of stretch.

**Fig. 2:**
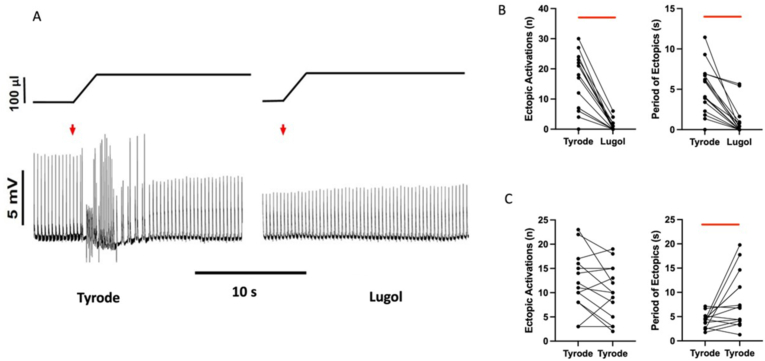
Lugol attenuates mechanically induced ectopics A. Upper trace, schematic of change in balloon volume, lower trace, pseudo-ECG, from a Langendorff perfused isolated rat heart in sinus rhythm. The red arrows indicates stretch of the LV by injection of 100 μL into a balloon placed in the LV lumen. Records from the same heart before (Tyrode) and after (Lugol) irrigation of the LV lumen with 0.1 mL Lugol (applied over a 10 s period, then Tyrode's washed). Mechanically-activated ectopics were evoked in the Tyrode's traces, disrupting the pseudo-ECG. In this heart, there were no mechanically-activated ectopics with Lugol and the heart beat rhythmically throughout stretch. B&C. Number of ectopics and period of ectopics for individual stretches under Tyrode's to Lugol conditions (B) and Tyrode's to Tyrode's (C). Lugol caused a significant reduction in both parameters, Tyrode's had no effect on the number of ectopics, while the period was significantly increased (see text). Data from 16 stretches in B&C. Bars indicate P < 0.05 (For interpretation of the references to colour in this figure legend, the reader is referred to the Web version of this article.)

In the presence of Tyrode's, stretch consistently provoked ectopic activations. However, following exposure to Lugol, ectopics were significantly attenuated, by 93%. Fig. 2A shows an example where, following brief endocardial exposure to Lugol, mechanically-induced arrhythmias were abolished. Fig. 2B shows data for individual stretches with a significant attenuation of both the number and period of ectopics following Lugol exposure. Following Lugol, ectopics were only seen in 6/16 stretches with –ve initial vectors only seen in 2 of these. The proportion of –ve going ectopics fell from 0.5 ± 0.1 to 0.3 ± 0.2 (P < 0.05. pairwise comparison with equivalent stretches in Tyrode's n = 6) When Tyrode's was used as the intervention, rather than Lugol, there was no effect on the number of ectopics, while the period of ectopics rose (Fig. 2C). The period was dependent upon the timing of the final ectopic (see Methods) which in some cases was a later, single ectopic within re-established sinus rhythm. Mean data are given in Table 1.

**Table 1:** Effects of Lugol and 9-Phenanthrol on mechanically induced ectopics and basal heart properties The left ventricle (LV) of isolated Langendorff perfused rat hearts were irrigated with Tyrode followed by irrigation with either Lugol (120 mM KI, 38.6 mM I_2_), 50 μM 9-Phenanthrol or Tyrode's. The LV was stretched pre and post intervention and the total number of ectopic activations and period of ectopics were measured. Lugol decreased both parameters, 9-Phen decreased ectopic number and Tyrode's increased ectopic period. Interventions had no significant effect on the heart rate or the increase in left ventricular developed pressure (Stretch LVDP) when balloon volume was increased from 50 μL to 150 μL. Data are mean ± SEM from 16 stretches for ectopics and 13–16 for LVDP, *p < 0.05. paired *t*-test Tyrode's vs Intervention.

N (hearts)		Lugol	9-Phen	Tyrode
		8	8	8
Number of ectopics	Tyrode	15.31 ± 2.32	11.27 ± 1.78	12.25 ± 1.41
	Intervention	1.0 ± 0.44 *	6.87 ± 2.11 *	9.94 ± 1.36

Period of ectopics (s)	Tyrode	5.02 ± 0.75	3.79 ± 0.74	4.21 ± 0.36
	Intervention	0.93 ± 0.47 *	2.94 ± 1.07	7.90 ± 1.38 *

Heart rate (bpm)	Tyrode	192 ± 8	207 ± 18	195 ± 12
	Intervention	209 ± 13	196 ± 13	180 ± 9

Stretch LVDP (mmHg)	Tyrode	48.5 ± 6.6	23.5 ± 3.9	24.8 ± 3.0
	Intervention	54.8 ± 7.1	27.7 ± 5.0	31.4 ± 4.6

### 3.2 Histological assessment of Lugol treatment and TRPM4 abundance

**Fig. 3:**
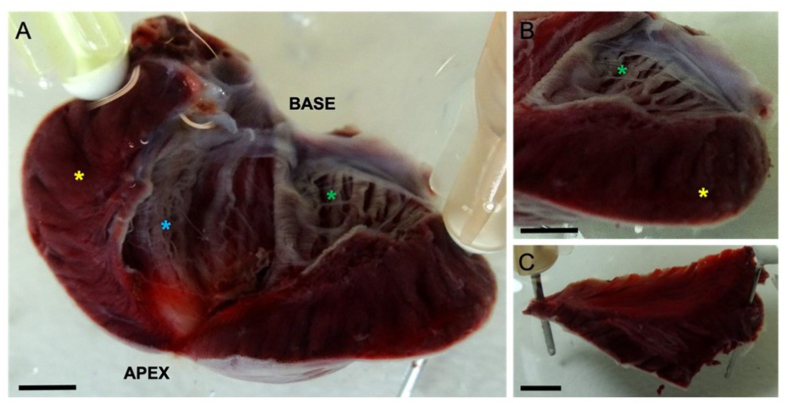
Necrotic tissue is restricted to the endocardial surface in Lugol treated hearts. The left ventricular (LV) lumen of isolated Langendorff perfused rat hearts were exposed to Lugol (120 mM KI, 38.6 mM I_2_) solution for 10 s, followed by Tyrode's wash. Hearts were stained with TTC and fixed in 10% formalin prior to imaging. (A) LV, with incision through the free wall. White, necrotic, tissue is restricted to the endocardial surface to a depth of approx. 200 μm. Prominent, ablated PFs are visible e.g. below the green asterick and on the septal wall. (B) Presence of necrotic tissue on the LV endocardial surface contrasts with its absence in both the LV myocardium and in (C), the endocardial surface of the untreated RV free wall taken from the same heart. (A&B) Yellow asterick denotes myocardium of the LV free wall, green and blue astericks denotes ablated tissue on the luminal surface of LV free wall and LV septal wall respectively. Scale bars, A–C: 2 mm. . (For interpretation of the references to colour in this figure legend, the reader is referred to the Web version of this article.)

**Fig. 4:**
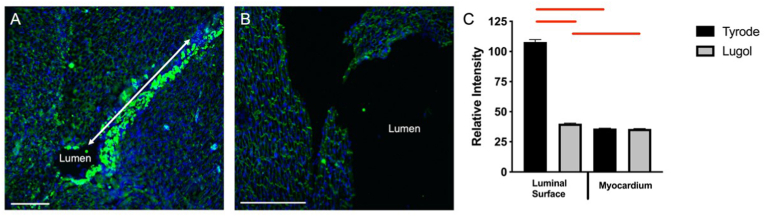
Lugol treatment attenuates endocardial connexion 40. The left ventricular (LV) lumen of isolated Langendorff perfused rat hearts were exposed to either (A) Tyrode's or (B) Lugol (120 mM KI, 38.6 mM I_2_) solution for 10 s, followed by Tyrode's wash. Cells were identified with DAPI stain (blue) and Purkinje fibres identified by dense connexin-40 staining (Cx40; bright green). (A) Dense Cx40 staining in the LV luminal surface of hearts exposed to Tyrode's only. In A the LV lumen is occluded in part of the image but follows the line of dense Cx40 staining indicated by the white arrow. (B) Absence of dense Cx40 stain on the LV luminal surface (Lumen) exposed to Lugol solution. (C) Relative intensity of Cx40 fluorescent signal at the luminal surface and deep myocardium, based upon the pixel gray value measured using Image J (Fiji). Data presented as mean ± SEM with one-way ANOVA performed in SPSS (P < 0.05). Tissue was imaged with an Evos Cell Imaging System. Scale bars, 250 μm. Data from n = 3 Tyrode treated hearts and n = 3 Lugol treated hearts. Bars indicate P < 0.05. (For interpretation of the references to colour in this figure legend, the reader is referred to the Web version of this article.)

Following treatment with Lugol, TTC staining was used to confirm that necrotic areas were limited to the LV endocardial surface, to a depth of approx. 200 μm, prominent ablation of PFs was visible (Fig. 3A and B). Necrotic tissue was not observed in untreated right ventricular tissue from the same hearts (Fig. 3C). To confirm an action of Lugol on PFs, high intensity Cx40 immunofluorescence (indicative of PFs) was found at the endocardial luminal surface and was significantly reduced by Lugol treatment (Fig. 4).

**Fig. 5:**
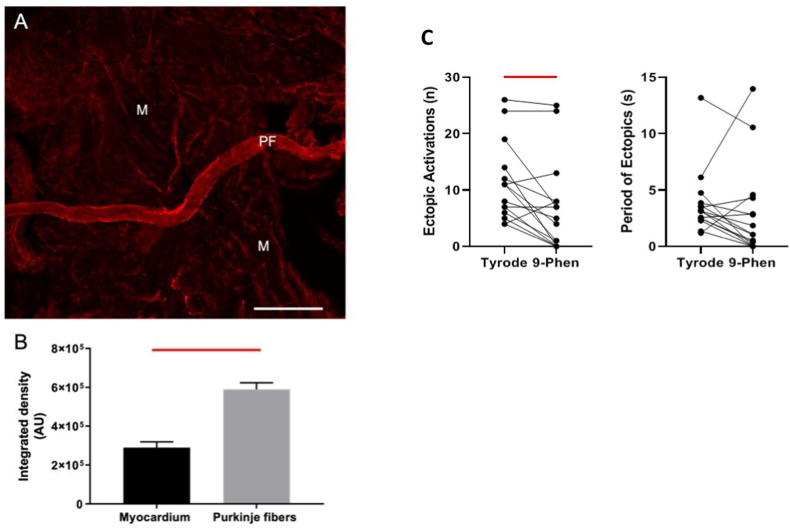
TRPM4 is more abundant in rat Purkinje fibres than myocardium and a TRPM4 blocker attenuates stretch-induced ectopics A. Purkinje Fibres (PF) and myocardium (M) were excised from the same area of rat LV free wall. (A) Tissue was immunolabelled for TRPM4 with primary antibody rabbit anti-TRPM4 (1:100; PA5-77324, Invitrogen) and secondary antibody goat anti-rabbit (1:200, Alexa 594. Thermofisher Scientific). Tissue was mounted in Prolong Gold (Thermofisher Scientific) on a glass slide. Two-dimensional images were acquired using an FV1000 Olympus confocal microscope. Increased TRPM4 immunofluorescence was observed in PF compared to M. Scale bar 250 μm. B. The integrated density and mean gray value of TRPM4 label was measured from myocardium and PFs (n = 3 hearts) using Image J-(Fiji) and presented as the mean integrated density ± SEM. Statistical analysis of data was performed in GraphPad Prism 7.03, and differences were compared using paired t-tests. The bar above the columns indicates (P < 0.05). C. Number of ectopics and period of ectopics for individual stretches under Tyrode to 50 μM 9-Phenanthrol (9-Phen) conditions in intact Langendorff hearts. 9-Phen (a TRPM4 blocker) caused a significant reduction in the number of ectopics. Data from 8 hearts and 15 stretches.Bars indicate P < 0.05. (For interpretation of the references to colour in this figure legend, the reader is referred to the Web version of this article.)

TRPM4 abundance was assessed with immunofluorescence. In agreement with studies from other species, we found a significantly greater abundance of TRPM4 in rat LV PFs than rat LV myocardium (Fig. 5A and B). LV luminal exposure to 9-Phen significantly decreased the number of ectopics but not their period (Fig. 5C). Mean data are given in Table 1. There was no effect of interventions on heart rate or LVDP at balloon volumes of 50 μl or 150 μl, indicating no effect on contractility (Table 1).

### 3.3 Activation in response to sinus and endomyocardial stimulation

**Fig. 6:**
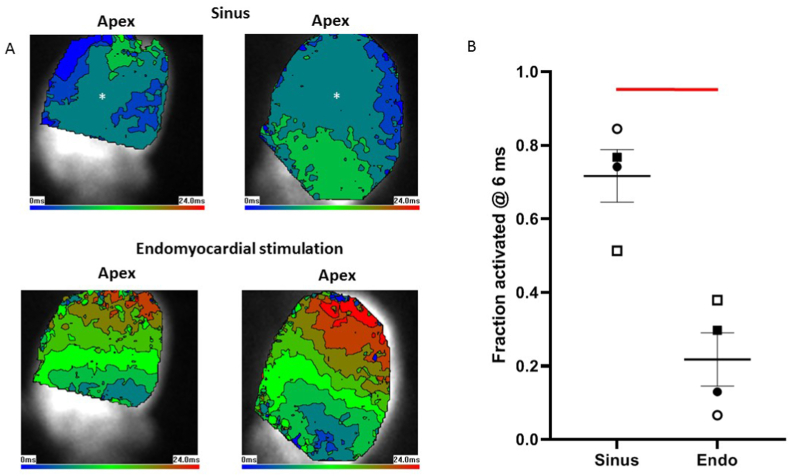
Activation from sinus stimulation spreads faster than from endomyocardial stimulation A. Optical mapping of left ventricular (LV) epicardial activation in 2 hearts in response to sinus stimulation (upper images) or focal endomyocardial stimulation near the LV base (lower traces). Images show colour coded 3 ms contours. Images are 80 × 80 pixels and 17.5 mm squares. In response to sinus stimulation, which involves conduction through the Purkinje network, there is a rapid spread of activation. In contrast focal endomyocardial stimulation results in a slower spread of activation. B. The fraction of epicardial surface activated at the 6 ms contour (colour identified by the asterix in A) in 4 hearts was significantly greater in response to sinus stimulation compared with endomyocardial stimulation. Thus the activation pattern can inform about the stimulation source. Hearts identified by symbol shape, individual data points and mean ± SEM shown (Bars indicate P < 0.05, paired *t*-test). . (For interpretation of the references to colour in this figure legend, the reader is referred to the Web version of this article.)

Information on the nature of electrical excitation can be derived from activation patterns (Martinez et al., 2018), we utilised this knowledge to further investigate stretch-induced ectopics. The principle is demonstrated in Fig. 6A which shows epicardial activation maps from 2 hearts. In these hearts, in response to sinus stimulation (which involves PF conduction), activation began apically and laterally and spread quickly over the surface of the epicardium. In contrast, in the same hearts, when activation was caused by focal stimulation of the basal endomyocardium the breakthrough was more localised (less blue colouration) and spread more slowly (brown and red colouration). The fraction of the epicardial surface activated at the 6 ms contour line is given for 4 hearts in Fig. 6B, there was a significantly greater activated fraction in response to sinus stimulation. Thus, different stimulation pathways produce distinct activation patterns.

### 3.4 Activation in ectopic excitations

**Fig. 7:**
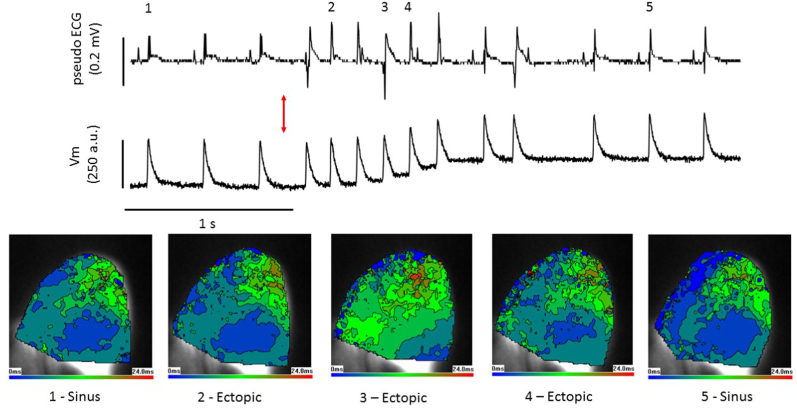
Stretch-induced ectopic activation mimics sinus and focal stimulation Pseudo-ECG (upper trace), optically mapped action potentials (middle trace) and epicardial activation maps, as described in Fig. 6 (lower images), in the left ventricle (LV) of a Langendorff rat heart. At the red arrow the LV is dilated from 50 μL to 150 μL by inflation of an indwelling balloon over a 2 s period, triggering ectopics. Activation maps from selected points (1–5) show sinus stimulations at 1&5 with typical rapid activation spread. Excitations 2&4 are ectopic but have activation patterns that closely mimic the sinus pattern, indicating the involvement of the Purkinje network. Excitation 3 is ectopic with activation spreading more slowly from a more basal site. Stretch-induced ectopics are multi-focal (see Discussion). . (For interpretation of the references to colour in this figure legend, the reader is referred to the Web version of this article.)

**Fig. 8:**
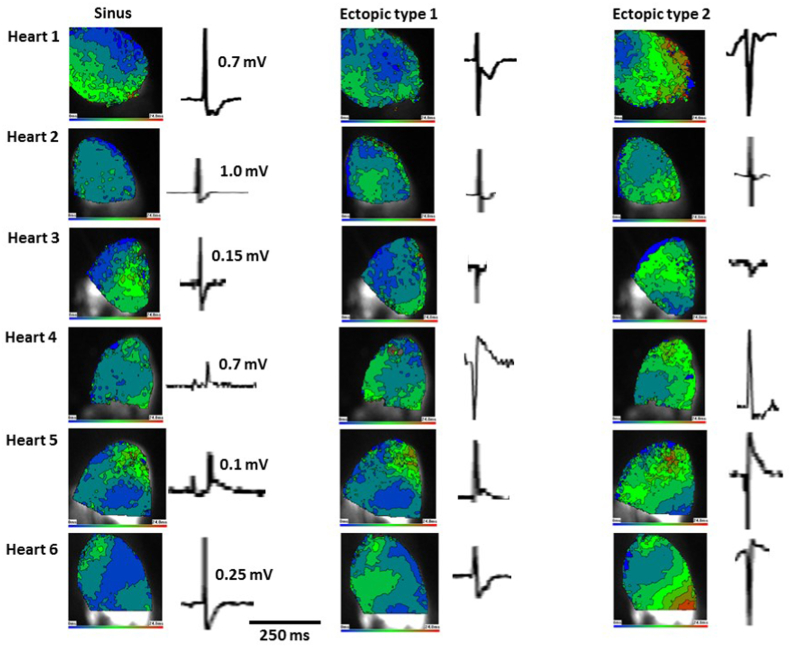
Stretch-induced ectopic activations Epicardial activation in 6 rat left ventricles (as described in Fig. 6). For each heart an example of activation in response to sinus stimulation and stretch-induced ectopics are shown with the associated pseudo-ECG waveform. The amplitude of the sinus R-wave is given as a voltage reference. Sinus and ectopic type 1 activations spread rapidly while ectopic type 2 spread more slowly. Positive and negative going pseudo-ECG waveforms occur with both type of activation pattern. For each heart both ectopic types were taken from the same stretch (see Fig. 7). Varied activations within a single run of dilution-induced ectopics are common. (For interpretation of the references to colour in this figure legend, the reader is referred to the Web version of this article.)

The same heart orientation (apex to top) and contour colour coding is used in Fig. 6, Fig. 7, Fig. 8. Fig. 7 shows the pseudo-ECG and optically measured action potentials during a LV stretch experiment. Increase in balloon volume from 50 μL to 150 μL led to a brief period of ectopic activity followed by a return to sinus rhythm. The shift in the baseline of the optical signal was approximately 10^3^ times slower than the upstroke of the action potential and had minimal effect on activation mapping. Epicardial activations at 5 selected points are shown. Activations 1 and 5 represent sinus activations with initial breakthrough, in this heart, occurring in the basal-medial region. Activations 2 and 4 were identified as ectopics, from the pseudo-ECG but had activation patterns matching sinus activations, strongly indicating a PF-network involvement. Activation 3 was also ectopic, with a negative-going initial vector. Here activation was more localised in the base and spread more slowly than in the other 4 activations, such a pattern might arise from a small region of endomyocardial stimulation or involvement of a restricted part of the PF-network. Similar observations were made in 5 other hearts. Fig. 8 gives examples of sinus activation and ectopic activation with rapid (labelled type1) or more slowly spreading activation (labelled type2) profiles in each of the 6 hearts. There was no overall dominant pattern and an intermingling of sinus-like and non-sinus like ectopic activations (see Discussion).

## 4 Discussion

### 4.1 Main findings

The main findings of our study are that, in isolated rat hearts the endocardial region plays an important role in acute stretch/dilation-induced ectopics, indicating the importance of regional stimulation in mechanically-induced arrhythmias. The PF network is involved in these ectopics, indicating tissues other than the myocardium can be associated with ventricular mechanically-induced arrhythmias. Contribution from the endomyocardium is not excluded, indicating complex interactions in the multifocal ectopics observed. We also find that, in line with other species previously tested, TRPM4 abundance is greater in PFs than myocardium, strengthening evidence that TRPM4 has a greater influence in conductive tissue than myocardium. Additionally, a TRPM4 blocker reduced the number of ectopics, giving potential mechanistic insight.

### 4.2 Endocardial site of action

Irrigation of the LV lumen was used to target the endocardial surface with agents. An endocardial, rather than bulk myocardial, site of action was expected because a steep fall in agent concentration would occur between the LV lumen and the myocardium. This is due to the physical presence of the endothelium and limitations to diffusion between the LV lumen and myocardial tissue also Langendorff perfused with Tyrode's, via the aorta and coronary circulation. TTC staining confirmed that the necrotic effect of brief exposure to Lugol was confined to the endocardial surface, in agreement with previous studies regarding the action of Lugol (Damiano et al., 1986; Dosdall et al., 2008; Myles et al., 2012; Chen et al., 1993; Cha et al., 1995).

Furthermore, while both Lugol and 9-Phen significantly attenuated the number of mechanically-induced ectopics, neither affected LVDP, indicating little, if any, effect on the bulk myocardium. We conclude that stimulation of the endocardial region plays an important role in these ectopics and that the bulk myocardium is not their major source. Regional or local stimulation may be a common factor in mechanically-induced arrhythmias e.g. (Quinn et al., 2017) reported focal mechanical deformation of the epicardial surface, triggered ectopics that arose from the site of mechanical stimulation.

### 4.3 Pharmacological and histological evidence to distinguish between tissues

Several tissue types, present at the endocardial surface, might contribute to ectopic mechanisms. Damage to the endocardial endothelium is known to affect the underlying myocardium (Brutsaert et al., 1996) and mechanical stimulation of the endocardial endothelium has been reported (Calaghan and White, 2001). However, the modulation of the myocardium by the endocardial endothelium is paracrine in nature and too slow (Brutsaert et al., 1996) to account for the rapid effects of stretch on electrical activity. Ectopics occurred early in the stretch period then spontaneously stopped, whilst a mechanism based on paracrine agents would be expected to be greater at the end rather than at the start of the stretch. Though gap junctions between endothelial cells and myocytes have been identified, their function appears to be associated with remodelling rather than acute modulation of cardiac electrophysiology (Johnson and Camelliti, 2018).

For an exclusively endomyocardial mechanism to explain our observations, a precise volume of endomyocardium, large enough to overcome sink-source effects of the bulk myocardium but small enough not to impact contractility when ablated or inhibited, would need to be excited. This volume would also need to generate ectopic activation patterns that mimic the particular sinus activation in a given heart, whilst by-passing the PF conduction network. Our data more credibly describe mechanisms that are dependent upon both PFs and the endomyocardium, for reasons that will now be discussed.

An extensive, PF network on the endocardial surface has been identified in rat hearts e.g. (Bordas et al., 2010) and because of their thin structure and surface location, PFs are particularly susceptible to agents introduced into the LV lumen. The near 1D cable properties of thin PFs reduce sink-source issues associated with electrical activation and conduction, when compared with the myocardium (Haissaguerre et al., 2016). Lugol has not previously been used to modulate electrical activity associated with mechanical stimulation, it is however, an established tool for the preferential ablation of PFs and modulation of electrical activity (Damiano et al., 1986; Dosdall et al., 2008; Myles et al., 2012; Chen et al., 1993; Cha et al., 1995). We saw clear evidence of necrotic PFs and a reduction of Cx40 rich structures i.e. PFs (Atkinson et al., 2013) at the LV luminal surface.

Lugol reduced ectopics by 93%, a significant reduction in the proportion of remaining ectopics displaying –ve initial vectors may represent greater access of Lugol to freewall sites distal to the apical ECG electrode (see section 4.4). In summary Lugol ablated PFs and attenuated ectopics with sinus-like activation, providing strong evidence for involvement of PFs in these ectopics.

The TRPM4 blocker, 9-Phen, partially reduced mechanically-activated ectopics. In species where the comparison has been made, TRPM4 is more abundant in conductive tissue than ventricular tissue (Guinamard et al., 2015), our findings now extend this observation to rat. In rabbit, TRPM4 single channel activity was reported in PFs but not in ventricular myocytes and 9-Phen modulated the action potential of PFs but not ventricular myocytes (Hof et al., 2016). Unless the pharmacology of TRPM4 channels in rats is distinct from other rodents, we conclude that, at least some of the action of 9-Phen is via effects on PFs. 9-Phen may target stretch-activation of TRPM4 channels, reported in arteries (Li et al., 2014; Earley et al., 2007) but as yet unstudied in cardiac PFs. Alternatively TRPM-4 blockade may affect electrical activity by a generic decrease in excitability which increases the threshold for the triggering and/or sustaining of arrhythmias (Guinamard et al., 2015).

### 4.4 Profile of stretch-induced arrhythmias in rat hearts

Our observation of trains of ectopic excitations of varied form, beginning early during stretch and then self-terminating, is similar to the pattern of ectopics provoked by balloon inflation in rabbit LV, described by (Dick and Lab, 1998). We found the source of electrical excitation influenced the pattern of activation recorded on the epicardial surface, as previously described (Martinez et al., 2018). Sinus stimulation includes conduction through the PF network and in response to sinus stimulation, activation patterns were initially broader and faster to spread than focal stimulation of the endomyocardium.

Ectopic pseudo-ECG and optically measured epicardial activation revealed multifocal activations that matched both sinus-like and focal formats within the same train. Ectopics with a slow spread of epicardial activation may originate from either the endomyocardium directly or discrete branches of the PF network distinct from the broader activation pattern of normal sinus rhythm. Positive going pseudo-ECGs being proximal and negative going being distal to the sinus His-PF conduction pathway.

However, activations with a positive going pseudo-ECG and fast spread of epicardial activation likely involve the proximal PF network, in or near to the His bundle, enabling the global sinus rhythm pattern to be closely mimicked. Fast activations with negative going pseudo-ECG waveforms (Hearts 3 & 4, Fig. 8) may reflect PF conduction from substantial PF branches distal to the sinus His-PF pathway. An endomyocardial source, in close proximity, could initially activate the PF network e.g. (Blackwell et al., 2022), but in either scenario PF conduction is required to create the observed activation pattern.

Overall we conclude that the mixed nature of the ectopic profiles suggests multiple sources and mechanisms are likely to be involved rather than a single ectopic site and mechanism, common to all hearts.

### 4.5 Study limitations

Arrhythmias may have been sustained by re-entry or by focal source mechanisms (which are dominant in multifocal morphologies). Our current observations do not distinguish between these mechanisms. Lugol did not abolish ectopics in all preparations, which might be due to incomplete ablation of the endocardial region or indicate that a few ectopics may arise or be sustained remote from the endocardial region. 9-Phen was reported to target late Na^+^ current in some pathologies, though it showed little effect in normal tissue (Hou et al., 2018). The conditions that enhanced late Na^+^ did not exist in our experiments.

### 4.6 Physiological implications

Whilst we have implicated PFs in acute stretch/dilation-induced ectopics, we do not propose this as a universal mechanism for mechanically-induced arrhythmias. For example, in *commotio cordis*, it is stimulation of the epicardial myocardium that triggers ectopics (Quinn and Kohl, 2021).

Although the Langendorff balloon-inflation model does not strive to replicate a specific pathology, arrhythmias are associated with acute ventricular dilation in mitral regurgitation (Basso et al., 2019) and in Tako tsubo cardiomyopathy (Lyon et al., 2021). More generally, stretch-activated ectopics, are relevant to several pathologies (Orini et al., 2017; Quinn and Kohl, 2021). PFs too are recognised as an important factor in clinical arrhythmias (Haissaguerre et al., 2016) with their ablation a treatment strategy (Samo Ayou et al., 2014). Mechanical stimulation and PFs may interact in pathological settings where mechanical and electrical heterogeneities within and between PFs, Purkinje-muscle junctions and myocardial tissue may be increased (Wiedmann et al., 1996; Walton et al., 2014). For example, across a border between healthy and ischemic (Ferrier, 1976) or infarcted/scar regions (Sinha et al., 2009) where already enhanced electrical heterogeneities (Haissaguerre et al., 2016) may be augmented by local systolic PF or myocardial lengthening of the compromised tissue.

### 4.7 Conclusions

The endocardial region is important in acute stretch/dilation-induced ectopics and PFs play a role in these arrhythmias.

## Funding statement

Supported by 10.13039/501100001537The University of Auckland, 10.13039/501100000780The European Union (10.13039/501100000654Marie Curie International Research Staff Exchange Scheme CORDIS-3D grant to OB, EW) and 10.13039/501100000274British Heart Foundation grant PG/19/47/34335 to EW, OB, RW).

## CRediT authorship contribution statement

**Miriam Hurley:** Conceptualization, Investigation, Formal analysis, Writing – review & editing. **Sarbjot Kaur:** Investigation, Formal analysis, Writing – review & editing. **Richard Walton:** Conceptualization, Funding acquisition, Investigation, Formal analysis, Writing – review & editing. **Amelia Power:** Investigation, Formal analysis, Writing – review & editing. **Michel Haïssaguerre:** Formal analysis, Writing – review & editing. **Olivier Bernus:** Conceptualization, Funding acquisition, Investigation, Formal analysis, Writing – review & editing. **Marie-Louise Ward:** Conceptualization, Funding acquisition, Investigation, Formal analysis, Writing – review & editing. **Ed White:** Conceptualization, Funding acquisition, Investigation, Formal analysis, Writing – review & editing.

## Declaration of competing interest

The authors declare that they have no known competing financial interests or personal relationships that could have appeared to influence the work reported in this paper.

## Data Availability

Data will be made available on request.

## References

[bib1] Atkinson A., Inada S., Li J., Tellez J.O., Yanni J., Sleiman R., Allah E.A., Anderson R.H., Zhang H., Boyett M.R., Dobrzynski H. (2011). Anatomical and molecular mapping of the left and right ventricular His-Purkinje conduction networks. J. Mol. Cell. Cardiol..

[bib2] Atkinson A.J., Logantha S.J., Hao G., Yanni J., Fedorenko O., Sinha A., Gilbert S.H., Benson A.P., Buckley D.L., Anderson R.H., Boyett M.R., Dobrzynski H. (2013). Functional, anatomical, and molecular investigation of the cardiac conduction system and arrhythmogenic atrioventricular ring tissue in the rat heart. J. Am. Heart Assoc..

[bib3] Basso C., Iliceto S., Thiene G., Perazzolo Marra M. (2019). Mitral valve prolapse, ventricular arrhythmias, and sudden death. Circulation.

[bib4] Bell R.M., Mocanu M.M., Yellon D.M. (2011). Retrograde heart perfusion: the Langendorff technique of isolated heart perfusion. J. Mol. Cell. Cardiol..

[bib5] Benoist D., Stones R., Benson A.P., Fowler E.D., Drinkhill M.J., Hardy M.E., Saint D.A., Cazorla O., Bernus O., White E. (2014). Systems approach to the study of stretch and arrhythmias in right ventricular failure induced in rats by monocrotaline. Prog. Biophys. Mol. Biol..

[bib6] Bers D.M. (2002). Cardiac excitation-contraction coupling. Nature.

[bib7] Blackwell D.J., Faggioni M., Wleklinski M.J., Gomez-Hurtado N., Venkataraman R., Gibbs C.E., Baudenbacher F.J., Gong S., Fishman G.I., Boyle P.M., Pfeifer K., Knollmann B.C. (2022). The Purkinje-myocardial junction is the anatomic origin of ventricular arrhythmia in CPVT. JCI Insight.

[bib8] Bode F., Sachs F., Franz M.R. (2001). Tarantula peptide inhibits atrial fibrillation. Nature.

[bib9] Bordas R., Grau V., Burton R.B., Hales P., Schneider J.E., Gavaghan D., Kohl P., Rodriguez B. (2010). Integrated approach for the study of anatomical variability in the cardiac Purkinje system: from high resolution MRI to electrophysiology simulation. Annu Int Conf IEEE Eng Med Biol Soc.

[bib10] Boyden P.A., Dun W., Robinson R.B. (2016). Cardiac Purkinje fibers and arrhythmias; the GK moe award lecture 2015. Heart Rhythm.

[bib11] Brutsaert D.L., De Keulenaer G.W., Fransen P., Mohan P., Kaluza G.L., Andries L.J., Rouleau J.L., Sys S.U. (1996). The cardiac endothelium: functional morphology, development, and physiology. Prog. Cardiovasc. Dis..

[bib12] Calaghan S.C., White E. (2001). Contribution of angiotensin II, endothelin 1 and the endothelium to the slow inotropic response to stretch in ferret papillary muscle. Pflügers Archiv.

[bib13] Canale E., Campbell G.R., Uehara Y., Fujiwara T., Smolich J.J. (1983). Sheep cardiac Purkinje fibers: configurational changes during the cardiac cycle. Cell Tissue Res..

[bib14] Cha Y.M., Uchida T., Wolf P.L., Peters B.B., Fishbein M.C., Karagueuzian H.S., Chen P.S. (1995). Effects of chemical subendocardial ablation on activation rate gradient during ventricular fibrillation. Am. J. Physiol..

[bib15] Chen P.S., Wolf P.L., Cha Y.M., Peters B.B., Topham S.L. (1993). Effects of subendocardial ablation on anodal supernormal excitation and ventricular vulnerability in open-chest dogs. Circulation.

[bib16] Damiano R.J., Smith P.K., Tripp H.F., Asano T., Small K.W., Lowe J.E., Ideker R.E., Cox J.L. (1986). The effect of chemical ablation of the endocardium on ventricular fibrillation threshold. Circulation.

[bib17] Dick D.J., Lab M.J. (1998). Mechanical modulation of stretch-induced premature ventricular beats: induction of mechanoelectric adaptation period. Cardiovasc. Res..

[bib18] Dominguez G., Fozzard H.A. (1979). Effect of stretch on conduction velocity and cable properties of cardiac Purkinje fibers. Am. J. Physiol..

[bib19] Dosdall D.J., Tabereaux P.B., Kim J.J., Walcott G.P., Rogers J.M., Killingsworth C.R., Huang J., Robertson P.G., Smith W.M., Ideker R.E. (2008). Chemical ablation of the Purkinje system causes early termination and activation rate slowing of long-duration ventricular fibrillation in dogs. Am. J. Physiol. Heart Circ. Physiol..

[bib20] Earley S., Straub S.V., Brayden J.E. (2007). Protein kinase C regulates vascular myogenic tone through activation of TRPM4. Am. J. Physiol. Heart Circ. Physiol..

[bib21] Ferrier G.R. (1976). The effects of tension on acetylstrophanthidin-induced transient depolarizations and aftercontractions in canine myocardial and Purkinje tissues. Circ. Res..

[bib22] Franz M.R., Cima R., Wang D., Profitt D., Kurz R. (1992). Electrophysiological effects of myocardial stretch and mechanical determinants of stretch-activated arrhythmias. Circulation.

[bib23] Guinamard R., Bouvagnet P., Hof T., Liu H., Simard C., Sallé L. (2015). TRPM4 in cardiac electrical activity. Cardiovasc. Res..

[bib24] Haïssaguerre M., Shah D.C., Jaïs P., Shoda M., Kautzner J., Arentz T., Kalushe D., Kadish A., Griffith M., Gaïta F., Yamane T., Garrigue S., Hocini M., Clémenty J. (2002). Role of Purkinje conducting system in triggering of idiopathic ventricular fibrillation. Lancet.

[bib25] Haissaguerre M., Vigmond E., Stuyvers B., Hocini M., Bernus O. (2016). Ventricular arrhythmias and the His-Purkinje system. Nat. Rev. Cardiol..

[bib26] Hof T., Sallé L., Coulbault L., Richer R., Alexandre J., Rouet R., Manrique A., Guinamard R. (2016). TRPM4 non-selective cation channels influence action potentials in rabbit Purkinje fibres. J. Physiol..

[bib44] Hou J.W., Fei Y.D., Li W., Chen Y.H., Wang Q., Xiao Y., Wang Y.P., Li Y.G. (2018). The transient receptor potential melastatin 4 channel inhibitor 9-phenanthrol modulates cardiac sodium channel. Br. J. Pharmacol..

[bib27] Johnson R.D., Camelliti P. (2018). Role of non-myocyte gap junctions and connexin hemichannels in cardiovascular health and disease: novel therapeutic targets?. Int. J. Mol. Sci..

[bib28] Kaufmann R., Theophile U. (1967). [Autonomously promoted extension effect in Purkinje fibers, papillary muscles and trabeculae carneae of rhesus monkeys]. Pflugers Arch. für Gesamte Physiol. Menschen Tiere.

[bib29] Kohl P., Sachs F., Franz M.R. (2011).

[bib30] Li Y., Baylie R.L., Tavares M.J., Brayden J.E. (2014). TRPM4 channels couple purinergic receptor mechanoactivation and myogenic tone development in cerebral parenchymal arterioles. J. Cerebr. Blood Flow Metabol..

[bib31] Lyon A.R., Citro R., Schneider B., Morel O., Ghadri J.R., Templin C., Omerovic E. (2021). Pathophysiology of takotsubo syndrome: JACC state-of-the-art review. J. Am. Coll. Cardiol..

[bib32] Martinez M.E., Walton R.D., Bayer J.D., Haïssaguerre M., Vigmond E.J., Hocini M., Bernus O. (2018). Role of the purkinje-muscle junction on the ventricular repolarization heterogeneity in the healthy and ischemic ovine ventricular myocardium. Front. Physiol..

[bib33] Myles R.C., Wang L., Kang C., Bers D.M., Ripplinger C.M. (2012). Local β-adrenergic stimulation overcomes source-sink mismatch to generate focal arrhythmia. Circ. Res..

[bib34] Orini M., Nanda A., Yates M., Di Salvo C., Roberts N., Lambiase P.D., Taggart P. (2017). Mechano-electrical feedback in the clinical setting: current perspectives. Prog. Biophys. Mol. Biol..

[bib35] Quinn T.A., Jin H., Lee P., Kohl P. (2017). Mechanically induced ectopy via stretch-activated cation-nonselective channels is caused by local tissue deformation and results in ventricular fibrillation if triggered on the repolarization wave edge (commotio cordis). Circ Arrhythm Electrophysiol.

[bib36] Quinn T.A., Kohl P. (2021). Cardiac mechano-electric coupling: acute effects of mechanical stimulation on heart rate and rhythm. Physiol. Rev..

[bib37] Samo Ayou R., Steen T., Agarwal S. (2014). Successful ablation of idiopathic ventricular fibrillation by targeting Purkinje potentials from right ventricle. Europace.

[bib38] Shiels H.A., White E. (2008). The Frank-Starling mechanism in vertebrate cardiac myocytes. J. Exp. Biol..

[bib39] Sinha A.M., Schmidt M., Marschang H., Gutleben K., Ritscher G., Brachmann J., Marrouche N.F. (2009). Role of left ventricular scar and Purkinje-like potentials during mapping and ablation of ventricular fibrillation in dilated cardiomyopathy. Pacing Clin. Electrophysiol..

[bib40] Walton R.D., Martinez M.E., Bishop M.J., Hocini M., Haïssaguerre M., Plank G., Bernus O., Vigmond E.J. (2014). Influence of the Purkinje-muscle junction on transmural repolarization heterogeneity. Cardiovasc. Res..

[bib41] White E., KOHL P., SACHS F., FRANZ M.R. (2005). Cardiac Mechano-Electric Feedback & Arrhythmias, from Patient to Pipette.

[bib42] Wiedmann R.T., Tan R.C., Joyner R.W. (1996). Discontinuous conduction at Purkinje-ventricular muscle junction. Am. J. Physiol..

[bib43] Yamazaki M., Mironov S., Taravant C., Brec J., Vaquero L.M., Bandaru K., Avula U.M., Honjo H., Kodama I., Berenfeld O., Kalifa J. (2012). Heterogeneous atrial wall thickness and stretch promote scroll waves anchoring during atrial fibrillation. Cardiovasc. Res..

